# Flexible Epoxy Resins Formed by Blending with the Diblock Copolymer PEO-*b*-PCL and Using a Hydrogen-Bonding Benzoxazine as the Curing Agent

**DOI:** 10.3390/polym11020201

**Published:** 2019-01-24

**Authors:** Wei-Chen Su, Fang-Chang Tsai, Chih-Feng Huang, Lizong Dai, Shiao-Wei Kuo

**Affiliations:** 1 Department of Materials and Optoelectronic Science, Center of Crystal Research, National Sun Yat-Sen University, Kaohsiung 804, Taiwan; d023100006@student.nsysu.edu.tw; 2 Hubei Key Laboratory of Polymer Materials, Key Laboratory for the Green Preparation and Application of Functional Materials (Ministry of Education), Hubei Collaborative Innovation Center for Advanced Organic Chemical Materials, School of Materials Science and Engineering, Hubei University, Wuhan 430062, China; 3 Department of Chemical Engineering, National Chung Hsing University, 145 Xingda Road, Taichung 402-27, Taiwan; huangcf@dragon.nchu.edu.tw; 4 Department of Material Science and Engineering, Fujian Provincial Key Laboratory of Fire Retardant Materials, College of Materials, Xiamen University, Xiamen, Fujian 361005, China; lzdai@xmu.edu.cn; 5 Department of Medicinal and Applied Chemistry, Kaohsiung Medical University, Kaohsiung 807, Taiwan

**Keywords:** hydrogen bonding, epoxy, benzoxazine, block copolymer, toughness

## Abstract

In this study, we enhanced the toughness of epoxy resin by blending it with the diblock copolymer poly(ethylene oxide–*b*–ε-caprolactone) (PEO-*b*-PCL) with a benzoxazine monomer (PA-OH) as the thermal curing agent. After thermal curing, Fourier transform infrared spectroscopy revealed that intermolecular hydrogen bonding existed between the OH units of the epoxy–benzoxazine copolymer and the C–O–C (C=O) units of the PEO (PCL) segment. Differential scanning calorimetry and dynamic mechanical analysis revealed that the glass transition temperature and storage modulus of the epoxy–benzoxazine matrix decreased significantly upon increasing the concentration of PEO-*b*-PCL. The Kwei equation predicted a positive value of *q*, consistent with intermolecular hydrogen bonding in this epoxy–benzoxazine/PEO-*b*-PCL blend system. Scanning electron microscopy revealed a wormlike structure with a high aspect ratio for PEO-*b*-PCL as the dispersed phase in the epoxy–benzoxazine matrix; this structure was responsible for the improved toughness.

## 1. Introduction

Because epoxy resins possess excellent thermal and mechanical properties, good chemical resistance, ready processability, and excellent adhesion to many substrates, they have many applications in composites—for example, as encapsulates for semiconductor materials, surface coatings, adhesives, and painting materials [1,2,3]. Nevertheless, common epoxy resins are often brittle; therefore, much effort has been exerted to improve the flexibility of epoxy resins and to understand their structure–property relationships [4,5]. Two main factors that affect the flexibility of modified epoxy resins are the size distribution and concentration of the additive components. As a result, two major approaches have been developed to enhance the flexibility of epoxy resins: (i) adding a soft segment (e.g., siloxane unit) into the main structure of the epoxy resin [6,7], and (ii) blending with a polymer or nanofiller (e.g., clay [8,9], polyhedral oligomer silsesquioxane [10,11,12], carbon nanotubes [13,14], liquid rubber [15], poly(ethylene oxide) (PEO) [16], poly(ε-caprolactone) (PCL) [17]) as a toughness agent. Blending an epoxy resin with a homopolymer or nanofiller in the absence of specific interactions will, however, usually result in macrophase separation through reaction-induced phase separation [18].

The use of amphiphilic diblock copolymers to improve the flexibility of epoxy resins has recently been investigated widely [19,20,21,22,23,24]. These diblock copolymers typically feature individual epoxy-immiscible and -miscible block segments; for example, PCL and PEO segments have been added to obtain, through hydrogen bonding, nanostructures within epoxy resins [25,26,27]. Using PEO-*b*-PCL diblock copolymers to control specific interactions, the miscibility of epoxy resins can be improved and macrophase separation can be suppressed [26]. Similar self-assembled structures have also been obtained from thermoset resins (e.g., phenolic, polybenzoxazine) and block copolymers [28,29,30,31,32].

Polybenzoxazine derivatives are new thermosetting resins with excellent thermal and mechanical properties, low water absorption, and low surface free energies [33,34,35]. Similar to other thermosetting resins, benzoxazine resins also have the shortcoming of being brittle. Furthermore, the phenolic units produced during ring opening polymerization of benzoxazine monomers can react further with epoxy units at certain temperatures to form epoxy–benzoxazine copolymer matrices; it is not necessary to add any catalyst for epoxy thermal curing, because the phenolic units can perform this role for ring opening polymerization [36,37]. Strong intramolecular hydrogen bonding between the hydroxyl (OH) units and the nitrogen atoms strongly influences the properties of polybenzoxazines [30], but there is a drawback in that intermolecular hydrogen bonding can occur with other polymers after thermal curing. As a result, most epoxies and polybenzoxazines usually form immiscible or phase-separated blends with homopolymers (e.g., PCL or PEO [38,39]), and form miscible blends only with poly(*N*-vinylpyrrolidone) (PVP) as a result of strong hydrogen bonding [40].

**Figure 1:**
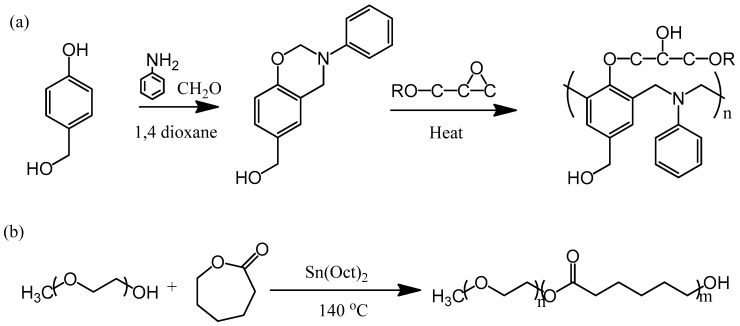
(**a**) Synthesis of the benzoxazine monomer PA-OH and a possible crosslinking structure for the epoxy resin. (**b**) Synthesis of the diblock copolymer PEO-*b*-PCL.

**Figure 2:**
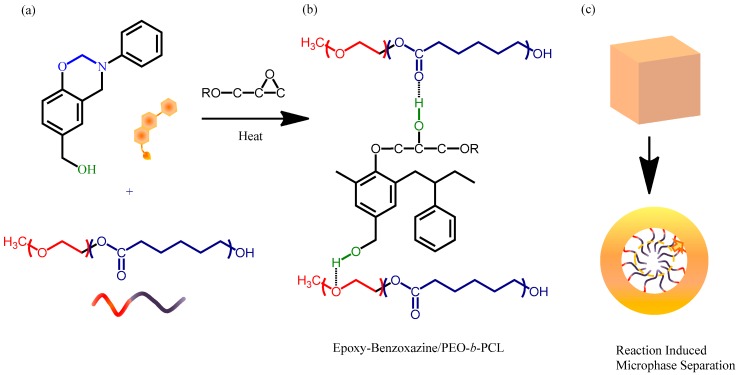
(**a**) Chemical structures of PA-OH and PEO-*b*-PCL. (**b**) Intermolecular hydrogen bonding between the OH units of the epoxy–benzoxazine (obtained after thermal curing) and the C–O–C units of the PEO segment and the C=O units of the PCL segment. (**c**) Self-assembled wormlike structures formed through reaction-induced microphase separation.

To maintain strong intramolecular hydrogen bonding within a polybenzoxazine, we wondered whether incorporating another OH group into the benzoxazine monomer would allow the formation of an additional set of hydrogen bonding interactions with other homopolymers, and thereby solve the miscibility problem. Previously, we synthesized the monomer PA-OH from 4-hydroxybenzyl alcohol, CH_2_O, and aniline (Figure 1). In this study, we prepared a flexible epoxy resin through blending with the diblock copolymer PEO-*b*-PCL and using this benzoxazine monomer as the curing agent (Figure 2). The OH units of the epoxy and the polybenzoxazine could form hydrogen bonds with the ether units of the PEO segment or the C=O units of the PCL segment. Herein, we discuss the hydrogen bonding interactions, miscibility, mechanical properties, and phase behavior of these systems.

## 2. Experimental Section

### 2.1. Materials

4-Hydroxybenzyl alcohol, CH_2_O, and aniline were purchased from Aldrich (St Louis, MO, USA). The (3-phenyl-3,4-dihydro-2*H*-1,3-benzoxazin-6-yl)methanol benzoxazine monomer (PA-OH; ^1^H NMR (CDCl_3_, ppm): 4.6 (s, CC**H**_2_N), 5.3 (s, NC**H**_2_O), 4.5 (s, ArC**H**_2_OH), 6.7–7.4 (m, Ar); ^13^C NMR (CDCl_3_, ppm): 79.5 (N**C**H_2_O), 50.5 (C**C**H_2_N), 64.9 (Ar**C**H_2_OH)) was synthesized using a previously reported procedure (Figure 1a) [30,41]. The PEO-*b*-PCL diblock copolymer (*M*_n_ = 15,000; PDI = 1.15) was synthesized through ring opening polymerization of ε-caprolactone, using monomethoxy-poly(ethylene glycol) (MPEG-5K) as the initiator and Sn(Oct)_2_ as the catalyst (Figure 1b). The diglycidyl ether of bisphenol A (DER 331, DGEBA) was obtained from Nan-Ya Chemical (Taipei, Taiwan).

### 2.2. Flexible Epoxy Resin

The epoxy resin, PA-OH (curing agent), and PEO-*b*-PCL (flexibility agent) were dissolved in THF at the desired ratio. After the solution had become homogenous, the solvent was evaporated slowly and the residue was vacuum-dried overnight at room temperature. The epoxy–benzoxazine resins were cured using the following temperature profile: 110 °C for 3 h, 160 °C for 2 h, 180 °C for 2 h, 200 °C for 1 h, 220 °C for 1 h, and 240 °C for 0.5 h (heating rate: 1 °C min^−1^).

### 2.3. Characterization

^1^H NMR spectra were measured using a Bruker AM 500 spectrometer (McKinley Scientific, Sparta, NJ, USA), with CDCl_3_ as the solvent and tetramethylsilane (TMS) as the external standard. The molecular weight and polydispersity of the PEO-*b*-PCL diblock copolymer were determined through gel permeation chromatography (GPC) using a Waters 510 high-performance liquid chromatography (HPLC) system and DMF as the eluent (flow rate: 0.6 mL min^−1^). The thermal behavior of the epoxy–benzoxazine resins was examined through differential scanning calorimetry (DSC) using a TA-Q20 instrument (TA Instrument, New Castle, DE, USA) operated over the temperature range from −90 to +240 °C under a N_2_ atmosphere (heating rate: 20 °C min^−1^). For nonisothermal crystallization experiments, the epoxy–benzoxazine resins with various amounts of PEO-*b*-PCL were first annealed at 240 °C for 5 min, and then the crystallization exotherm was obtained at temperatures down to −90 °C (cooling rate: 5 °C min^−1^). Fourier transform infrared (FTIR) spectra were measured using a Bruker Tensor-27 spectrophotometer (Billerica, MA, USA); 32 scans at a resolution of 4 cm^−1^ were collected using the KBr plate method. Field emission scanning electron microscopy (FE-SEM) was conducted using a JEOL JSM-7610F scanning electron microscope (JEOL, Tokyo, Japan); the samples were subjected to Pt sputtering for 2 min prior to measurement. The dynamic mechanical behavior of the samples was investigated using a PerkinElmer D8000 analyzer (PerkinElmer, Taipei, Taiwan); the cured samples were polished to approximately 30.0 × 13.0 × 3.0 mm^3^, and then the mechanical properties were determined at temperatures from −100 to +250 °C at a frequency of 1 Hz (heating rate: 2 °C min^−1^).

## 3. Results and Discussion

### 3.1. Analyses of PA-OH and PEO-b-PCL

**Figure 3:**
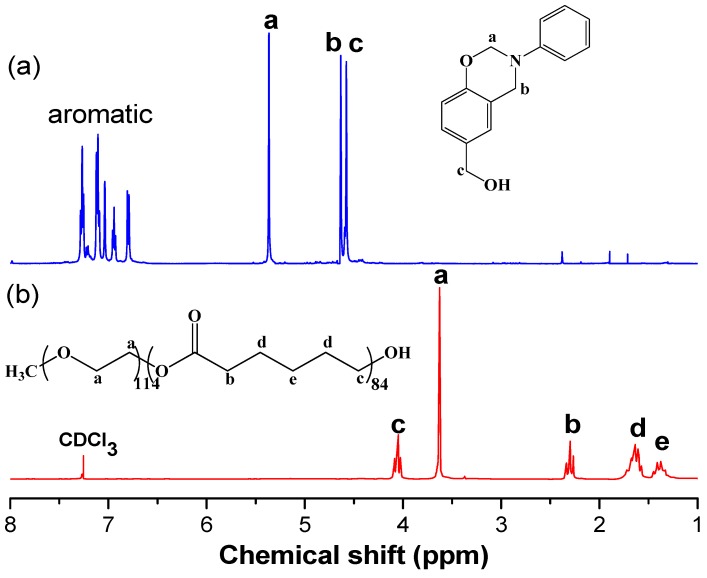
^1^H NMR spectra of (**a**) PA-OH and (**b**) PEO-*b*-PCL.

Figure 3 displays ^1^H NMR spectra of the benzoxazine monomer PA-OH and the diblock copolymer PEO-*b*-PCL used in this study. The ^1^H NMR spectrum of PA-OH (Figure 3a) features signals at 5.36 and 4.63 ppm, representing the CH_2_ protons of the oxazine ring; another at 4.55 ppm representing the C**H**_2_OH protons; and multiplets at 6.8–7.4 ppm, representing the aromatic protons. The molecular weight of PEO-*b*-PCL was measured based on its ^1^H NMR spectrum, considering the signals for the ether units of MPEG-5K at 3.65 ppm (–CH_2_CH_2_O–), and for the OCH_2_ units of the PCL segment at 4.10 ppm (*M*_n_ = 15,000; PDI = 1.15, based on GPC analysis).

### 3.2. Analyses of Epoxy–Benzoxazine/PEO-b-PCL Mixtures

**Figure 4:**
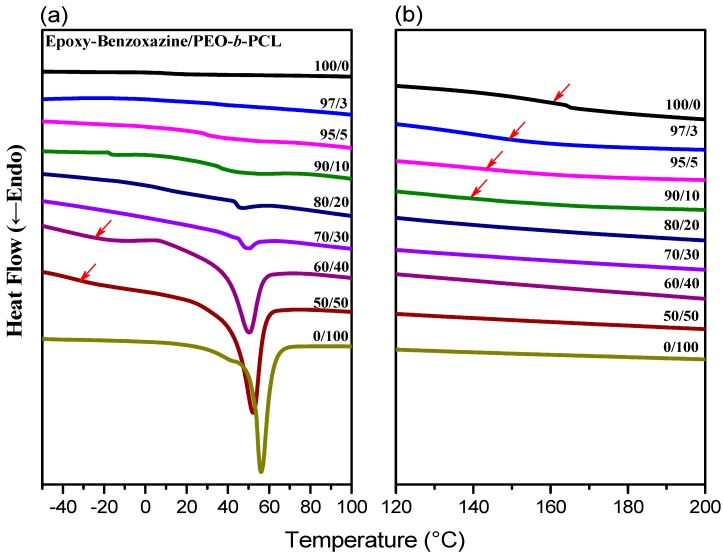
Differential scanning calorimetry (DSC) thermal analyses of epoxy–benzoxazine/PEO-*b*-PCL blends of various compositions (second heating scans), expanded from (**a**) −40 to +100 °C and (**b**) 120 to 200 °C.

DSC is generally applicable for determining the miscibility of polymer blend systems. Figure 4 displays the second heating scans of epoxy–benzoxazine/PEO-*b*-PCL blends of various compositions after thermal curing. For a 50:50 weight percentage blend of the epoxy and PA-OH (curing agent), the glass transition temperature was approximately 153 °C (Figure 4b). We used this composition to prepare blends featuring different amounts of PEO-*b*-PCL. The glass transition temperature of the epoxy–benzoxazine matrix decreased upon increasing the PEO-*b*-PCL composition, because of the lower glass transition temperature of the diblock copolymer. 

**Figure 5:**
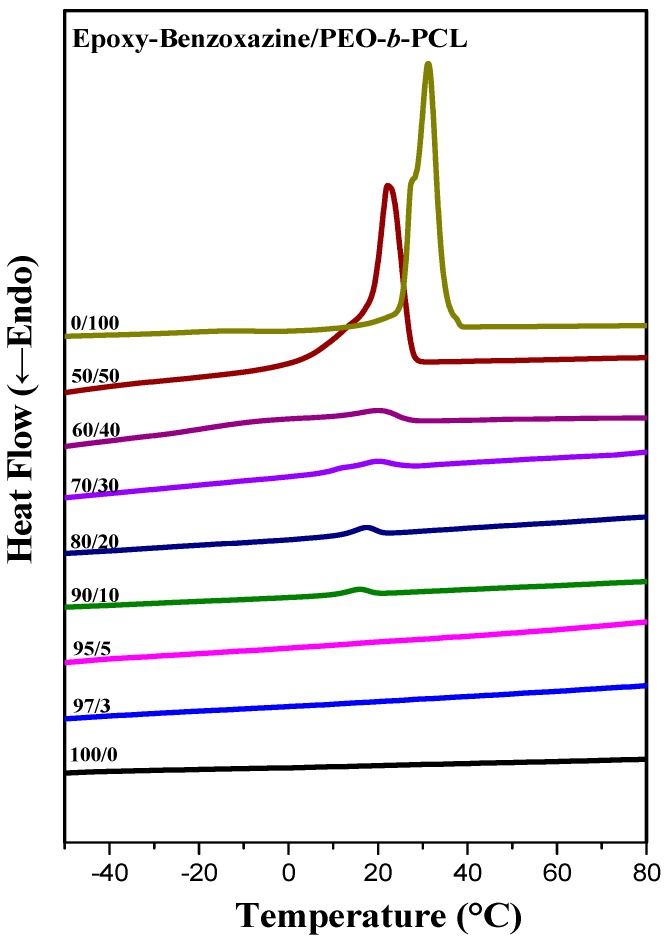
DSC cooling scans of epoxy-benzoxazine/PEO-*b*-PCL blends (cooling rate: 5 °C min^−1^).

Furthermore, the melting (Figure 4a) and crystallization (Figure 5) temperatures of the PCL and PEO block segments both decreased upon increasing the epoxy–benzoxazine concentration, due to hydrogen bonding interactions inducing thermodynamic and morphological effects. As a result, the melting temperatures for the PEO and PCL block segments were depressed in the presence of the epoxy–benzoxazine resin in this study. Because the glass transition temperatures of PCL and PEO are very close (*T*_g_ = −60 °C), the miscibility of PCL and PEO was difficult to determined based solely on the values of *T*_g_. We observed, however, that the values of *T*_g_ of PEO-*b*-PCL shifted to higher temperatures upon increasing the epoxy–benzoxazine concentrations. In general, benzoxazine/PEO and benzoxazine/PCL blends usually form phase-separated and immiscible systems because of the absence of strong hydrogen bonding in these blend systems; in this study, however, we used the benzoxazine PA-OH to introduce additional OH groups into the epoxy matrix to form intermolecular hydrogen bonds with the C=O units of PCL and the ether units of PEO (Figure 2b).

**Figure 6:**
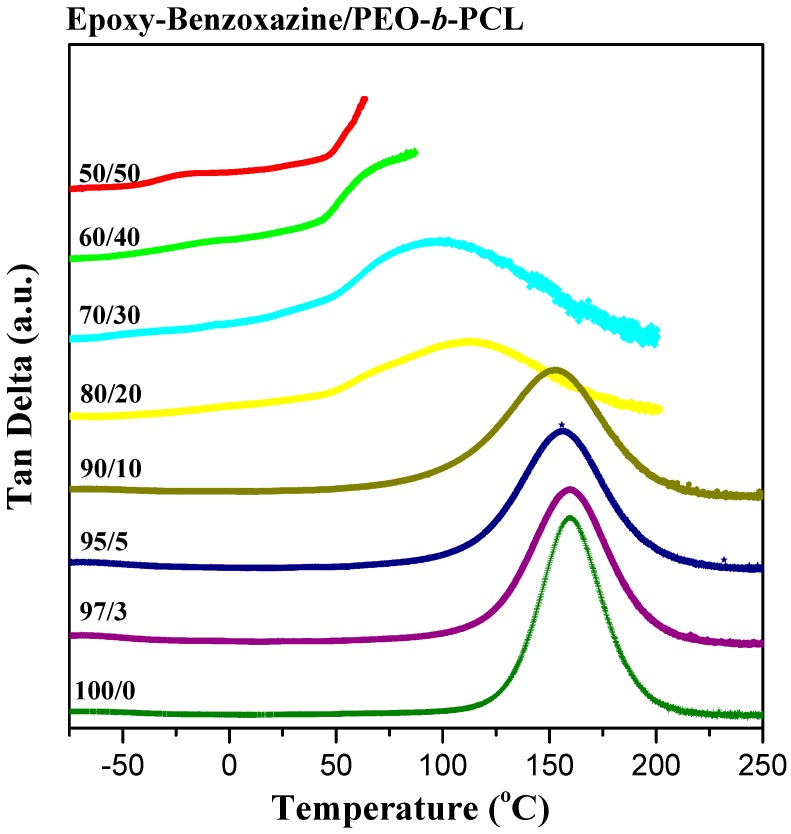
DMA tan δ–temperature curve of epoxy–benzoxazine/PEO-*b*-PCL blends (heating rate: 2 °C·min^−1^).

**Figure 7:**
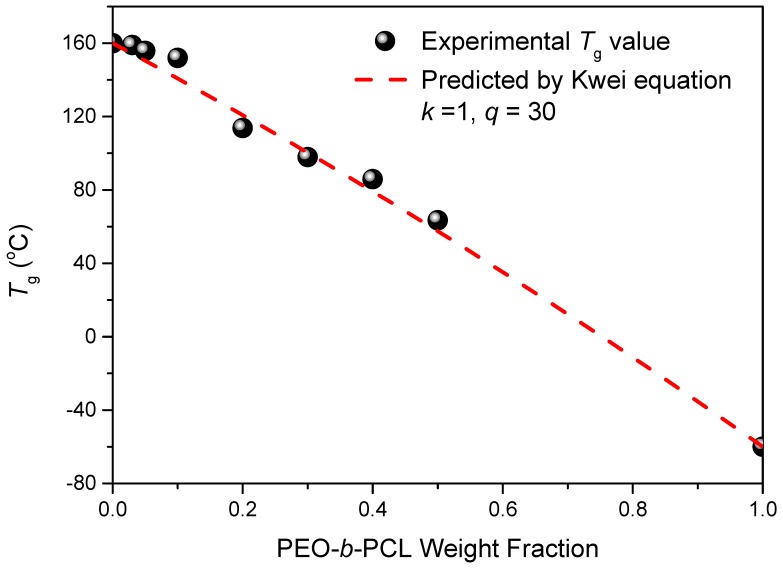
Glass transition temperature composition curve of epoxy–benzoxazine/PEO-*b*-PCL blends, based on the Kwei equation.

We used dynamic mechanical analyzer (DMA) to determine the glass transition behavior and mechanical properties of the epoxy–benzoxazine/PEO-*b*-PCL blend systems after thermal curing (Figure 6). The pure epoxy–benzoxazine thermosetting copolymer displayed a value of *T*_g_ of approximately 160 °C, based on its tan δ–temperature curve. The values of *T*_g_ determined using DMA were generally higher than those obtained through DSC analyses, as displayed in Figure 4. Nevertheless, similar to the results from the DSC analyses, the value of *T*_g_ of the epoxy–benzoxazine matrix decreased upon increasing the content of PEO-*b*-PCL because of the lower glass transition temperature of the diblock copolymer. Because hydrogen bonding presumably existed in this system, we used the Kwei equation to predict the values of *T*_g_ of the epoxy–benzoxazine copolymer blended with PEO-*b*-PCL [42]:

(1) $$T_{g}=\frac{W_{1}T_{g1}+kW_{2}T_{g2}}{W_{1}+kW_{2}}+qW_{1}W_{2}$$

where *W*_1_ is the weight fraction of PEO-*b*-PCL, *W*_2_ is the weight fraction of the epoxy–benzoxazine copolymer, *T*_g1_ is the glass transition temperature of PEO-*b*-PCL (both at −60 °C), *T*_g2_ is the glass transition temperature of the epoxy-benzoxazine copolymer (160 °C), and *k* and *q* are fitting constants. Figure 7 reveals that the values of *T*_g_ decreased upon increasing the concentration of PEO-*b*-PCL, suggesting that the flexibility might indeed be enhanced through this approach. In addition, we determined the values of *k* and *q* to be 1 and 30, respectively, based on Figure 7. The positive value of *q* indicates that, after thermal curing, the intermolecular hydrogen bonding between the OH units of the epoxy–benzoxazine copolymer and the ether and C=O groups of the PEO-*b*-PCL diblock copolymer was stronger than the self-association hydrogen bonding of the OH units of the epoxy–benzoxazine copolymer.

**Figure 8:**
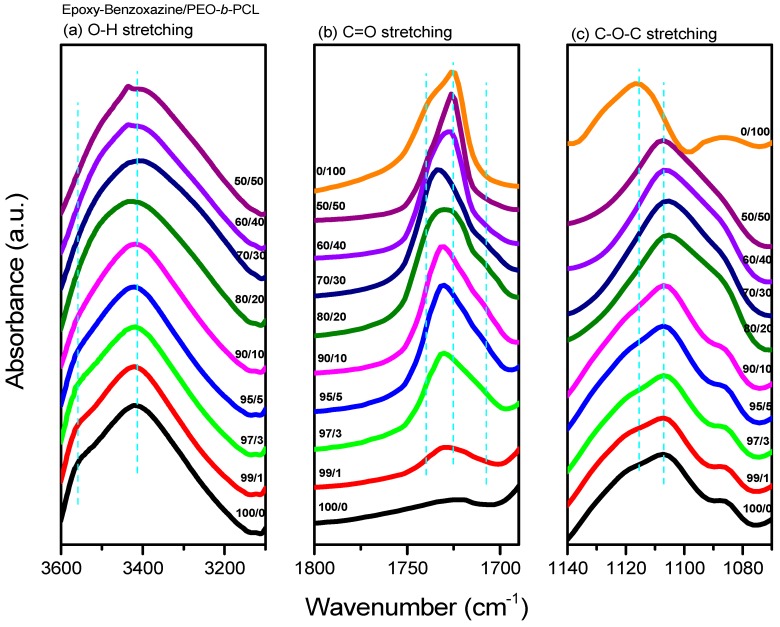
Fourier transform infrared (FTIR) spectra (recorded at room temperature) of epoxy–benzoxazine/PEO-*b*-PCL blends, displaying the (**a**) OH, (**b**) C=O, and (**c**) C–O–C stretching regions.

After thermal curing, FTIR spectroscopy (Figure 8) provided evidence for intermolecular hydrogen bonding between the OH units of the epoxy–benzoxazine copolymer and the ether or C=O groups of PEO-*b*-PCL. Figure 8a presents the OH stretching signals of the epoxy–benzoxazine/PEO-*b*-PCL blend system after thermal curing; these signals were sensitive to the presence of intermolecular hydrogen bonding. The spectrum of the pure epoxy–benzoxazine copolymer featured two major peaks: one representing the free OH units (at 3560 cm^−1^), and the other, a broad band at 3420 cm^−1^, representing the self-associated OH units. The intensity of the signal for the free OH units gradually decreased upon increasing the PEO-*b*-PCL concentration, while the broad band slightly shifted to 3435 cm^−1^, suggesting that the OH units of the pure epoxy-benzoxazine copolymer interacted with both the C=O units of the PCL segment and the ether units of the PEO segment. Furthermore, the signal for the C=O units of the PCL segment was also sensitive to the intermolecular hydrogen bonding, as revealed in Figure 8b. The spectrum of the pure PCL segment featured two signals for the C=O groups: one corresponding to the amorphous conformation or free C=O units (1734 cm^−1^) and the other to the crystalline conformation (1724 cm^−1^) [43]. The intensity of the signal for the crystalline peak gradually decreased upon increasing the content of the epoxy–benzoxazine copolymer, eventually disappearing at 80 wt %—similar to our observations from the DSC analyses. A signal for the hydrogen-bonded C=O units of the PCL segment appeared near 1708 cm^−1^ at relatively higher concentrations of the epoxy–benzoxazine copolymer. Thus, a higher fraction of OH groups provided a higher number of hydrogen-bonded C=O units, as expected. Figure 8c displays the ether stretching bands of the PEO segments; the spectrum of the pure PEO-*b*-PCL exhibited this absorption at 1116 cm^−1^, with this peak shifting to 1107 cm^−1^ upon increasing the concentration of the epoxy–benzoxazine copolymer [44,45], consistent with intermolecular hydrogen bonding occurring between the ether units of the PEO segment and the OH groups of the epoxy–benzoxazine copolymer after thermal curing (Figure 2b).

### 3.3. Mechanical Properties of Epoxy-Benzoxazine/PEO-b-PCL Mixtures

**Figure 9:**
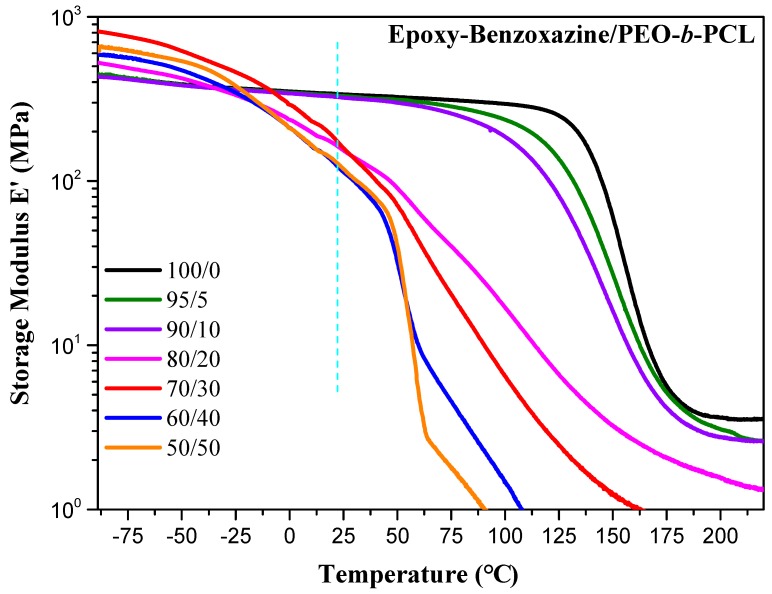
DMA storage modulus curve of epoxy–benzoxazine/PEO-*b*-PCL blends (heating rate: 2 °C·min^−1^).

The storage moduli of the epoxy-benzoxazine/PEO-*b*-PCL blends after thermal curing indicated (Figure 9) significantly improved toughness relative to that of PEO-*b*-PCL. The storage modulus deceased upon increasing concentration of PEO-*b*-PCL; it decreased significantly to 1.62 × 10^8^ Pa at 25 °C when the content of PEO-*b*-PCL was 20 wt %—recall that, at this composition, a significant decrease occurred in the value of *T*_g_: from 160 °C for the pure epoxy–benzoxazine copolymer to 113 °C. Bates et al. proposed that the nature of the self-assembled nanostructures affected the mechanical properties of a thermoset resin blended with a block copolymer [19]. 

**Figure 10:**
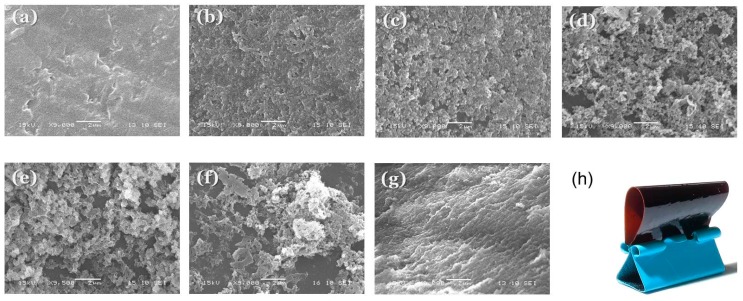
(**a**–**g**) Scanning electron microscope (SEM) images of epoxy–benzoxazine/PEO-*b*-PCL blends: (**a**) 100/0, (**b**) 95/5, (**c**) 90/10, (**d**) 80/20, (**e**) 70/30, (**f**) 60/40, (**g**) 50/50. (**h**) Photograph of a corresponding thin film (composition: 70/30).

Therefore, we used scanning electron microscopy (SEM) to investigate the morphologies and degrees of microphase separation in our epoxy–benzoxazine/PEO-*b*-PCL blends. We etched the thermosetting samples with NaOH to remove the PEO-*b*-PCL domains and maintain the epoxy–benzoxazine matrix. The pure epoxy–benzoxazine copolymer possessed a smooth surface morphology, as expected in the absence of PEO-*b*-PCL (Figure 10a). Upon increasing the PEO-*b*-PCL concentration, several porous structures were evident, with dimensions of approximately 100–200 nm at 5 and 10 wt % of PEO-*b*-PCL within the epoxy–benzoxazine copolymer matrix (Figure 8b,c). When the PEO-*b*-PCL content increased to 20 wt %, its domains changed from a dispersed phase to a semi-continuous phase, such that the PEO-*b*-PCL particles possessed an irregular wormlike structure (Figure 10d). Further increasing the PEO-*b*-PCL content to 30–50 wt % resulted in the epoxy–benzoxazine copolymer matrix changing from a continuous phase to a dispersed phase, with the particle size decreasing upon increasing the PEO-*b*-PCL concentration (Figure 8e–g). The toughening effect is known to depend significantly on the size and shape of the dispersed phase, and on the intermolecular interactions of the dispersed phase within the epoxy matrix; the wormlike micelle structure usually has a high aspect ratio, with an optimized length scale providing superior toughness over that of other self-assembled nanostructures, based on the toughness mechanism [19,20]. As a result, we conclude that the irregular wormlike structure of PEO-*b*-PCL dispersed in the epoxy–benzoxazine matrix (Figure 2c) was responsible for the improved toughness observed in our present DMA analyses. Figure 10h presents a flexible epoxy resin formed after blending with 30 wt % of PEO-*b*-PCL; this thin film displayed bendability and recoverable properties—features that are difficult to observe for pure epoxy and polybenzoxazine resins prepared through the synthesis method.

## 4. Conclusions

We have used DSC, FTIR spectroscopy, DMA, and SEM to investigate the hydrogen bonding interactions, miscibility, and phase behavior of epoxy–benzoxazine/PEO-*b*-PCL blends. FTIR spectra revealed evidence for intermolecular hydrogen bonding of the ether units of the PEO segments and the C=O units of the PCL segments with the OH groups of the epoxy–benzoxazine copolymer after thermal curing; this behavior was confirmed through DMA analyses and predictions made using the Kwei equation. In addition, the glass transition temperature (DSC) and storage modulus (DMA) of the epoxy–benzoxazine matrix both decreased significantly upon increasing the PEO-*b*-PCL concentrations. SEM revealed that a wormlike structure, with a high aspect ratio, for the PEO-*b*-PCL block copolymer as the dispersed phase in the epoxy–benzoxazine matrix was responsible for the improved toughness of the blend.
